# 

**Published:** 1906-08-04

**Authors:** 


					THE HOSPITAL. ' ? '
lye, i a
.1 r O.
August 4th, 1906.
1
f^037>Voi, XL. [*^p?] Saturday,
August 4th, 1906.
[3abBCereiPp!322Terms] PRICE THREEPENCE

				

